# A Magnetically Recoverable Fe_3_O_4_–NH_2_–Pd Sorbent for Capture of Mercury from Coal Derived Fuel Gas

**DOI:** 10.1038/s41598-017-07802-8

**Published:** 2017-08-07

**Authors:** Lina Han, Qinglian Li, Shuai Chen, Wei Xie, Weiren Bao, Liping Chang, Jiancheng Wang

**Affiliations:** 1 College of Materials Science and Engineering, Taiyuan University of Technology, Taiyuan, China; 2 State Key Laboratory Breeding Base of Coal Science and Technology Co-founded by Ministry of Science and Technology and Shanxi Province, Taiyuan University of Technology, Taiyuan, China; 3 Analytical Instrumentation Center, Institute of Coal Chemistry, Chinese Academy of Sciences, Taiyuan, China; 4 Chemical Engineering University of Newcastle, Callaghan, NSW 2308 Australia

## Abstract

A sort of magnetical material named Fe_3_O_4_–NH_2_–Pd was prepared by loading varying amounts of immobilizing Pd on the surface of the magnetic Fe_3_O_4_–NH_2_ microspheres. This magnetical material was used firstly for capturing Hg° from coal derived fuel gas based on its recoverability. The experimental results showed that the loading Pd on the amine-functionalized magnetite nanoparticles can greatly improve the efficiency of removing Hg° at a high temperature range between 200 and 300 °C. The magnetic Fe_3_O_4_–NH_2_–Pd sorbent with 5% Pd loaded exhibited significantly high activity and stability in capturing Hg°, affording over 93% capture efficiency at 200 °C for more than 8 hrs. Compared to the Fe_3_O_4_–NH_2_ sorbent that converted the Hg° as HgS, this Fe_3_O_4_–NH_2_–Pd sorbent can remove the Hg° by forming Pd-Hg amalgam and HgS. In addition, the experimental tests indicated that the as-synthesized Fe_3_O_4_–NH_2_–Pd sorbent still showed stable magnetic properties after two regeneration cycles in removing Hg°, which provided the opportunity for preparing a recyclable sorbent which can be easily separated and recovered for Hg° removal.

## Introduction

Coal gasification is a promising technology for coal cleaning utilization^1^. Some toxic gases, such as, H_2_S, HCl and Hg, may form during coal gasification. The environmental friendly utilization of the coal derived fuel gas requires the removal of these toxic gases, or converting the toxic gases to less toxic substance by performing catalyzed chemical reactions. The highly toxic metallic substance, mercury, is considered as a global threat not only because it may cause a significantly negative effect on human beings, it also may result in irreversibly environmental damage relating to its nature such as volatility, persistence and bioaccumulation^2–4^. Therefore, the control of mercury emissions from coal gasification arises a globally challenge. Overall, there are three types of mercury in the fuel gas generated from coal gasification. They are elemental mercury (Hg°), oxidized mercury (Hg^2+^, Hg^+^) and particle-bound-mercury (Hg^P^)^5^. Hg° is the predominant mercury that exists in the fuel gases because of the reducing environment during coal gasification^6, 7^. Hg° also is the most difficult substance to capture compared to others because it is highly vaporizable and nearly insoluble in water^8^. Therefore, this work will focus on the removal of the Hg°.

Previously, a number of sorbents have been used for Hg° removing in the literature. It has been found that the activated carbon and fly ash^9, 10^, particularly the activated carbon impregnated with sulfur^11, 12^, chlorine^13, 14^, iodine^15^ and bromine^16, 17^, showed a high efficiency in removing mercury. However, these previously studies also showed that the activated carbon and fly ash sorbents still suffer from practical utilization in separation and recycling besides the potential contamination caused by the converted products. Therefore, it has become crucial for preparing recyclable sorbents that could improve the efficiency in converting or removing mercury with the consideration of the economic and environmental impact. Recently, some work in the literature has showed that the magnetic nanoparticles (MNPs) were very promising as sorbent supports in promoting the mercury removal efficiency due to their large specific surface area and magnetic property^18, 19^. Granite *et al*. have reported that the supported noble metals, especially palladium, can be used for capturing mercury, arsenic, selenium and phosphorus from an experimental simulated fuel gases in an elevated temperatures range between 200 and 400 °C^20–24^.

Because of its practical importance, this field has also attracted considerably theoretical interests. Sasmaz *et al*. studied the adsorption of Hg on Pd binary alloys and overlays using PW91 functionally^25^. They found that Pd has the highest mercury binding energy in comparison to other noble metals. Lim *et al*. found that the number of vacancies surrounding the three-fold hollow site could affect the adsorption of Hg on Au surface^26^. It has been found that the adsorption performance of the sorbents can be improved by doping second metal or adding promoter to the sorbents. It was found that the addition of the small amounts of Au, Ag and Cu to the Pd could increase the overall mercury binding energy to the Pd surface^27^. DFT calculations were also carried out to investigate the adsorption of mercury and its compounds on the V_2_O_5_-WO_3_-TiO_2_, it is found that the ternary systems (V_2_O_5_-WO_3_-TiO_2_) showed a higher reactivity compared with the binary systems (V_2_O_5_-TiO_2_ or WO_3_-TiO_2_)^28^. Therefore, it is expected that the ideal mercury removal sorbents can be prepared by introducing the magnetic nanoparticles to the Pd sorbent to form a bimetallic or alloy sorbent.

The objectives of this work are to develop a recyclable sorbent for Hg removal. To achieve the goals, The metallic Pd and a magnetic material were selected as the active metal and support to prepare these sorbents. The performance of these sorbents in removing Hg° from the simulated fuel gas was investigated using a laboratory-scale fixed-bed reactor. These include: (1) the effects of temperature on the removal of Hg° of the as-synthesized Fe_3_O_4_–NH_2_ and Fe_3_O_4_–NH_2_–Pd sorbents; (2) the regeneration performance of the Fe_3_O_4_–NH_2_–Pd sorbent. The fresh and used Fe_3_O_4_–NH_2_ and Fe_3_O_4_–NH_2_–Pd sorbents were characterized by transmission electron microscopy (TEM), scanning electron microscopy (SEM), X-ray powder diffraction (XRD) and X-ray photoelectron spectroscopy (XPS). The mechanisms of Hg° removal over these sorbents were clarified based on the experimental results.

## Results and Discussion

### Effect of Pd loading content on Hg° removal over Fe_3_O_4_–NH_2_–Pd sorbents

**Figure 1:**
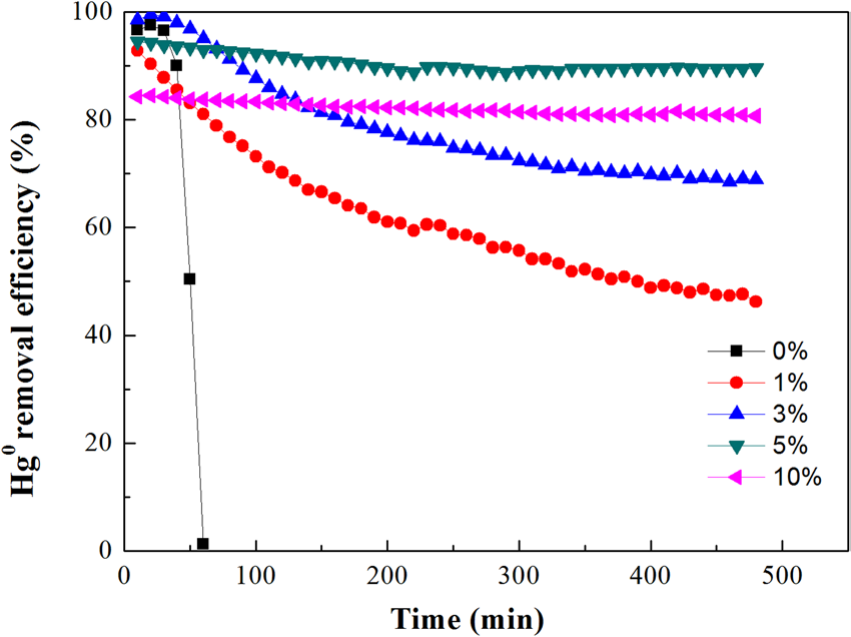
Efficiencies of Hg^0^ removal over Fe_3_O_4_–NH_2_–Pd sorbent with different contents of Pd at 200 °C.

A comparison of the Hg° removal efficiencies over Fe_3_O_4_–NH_2_ and Fe_3_O_4_–NH_2_–Pd sorbents at 200 °C according to our previous experimental results are shown in Fig. 1. For Fe_3_O_4_–NH_2_, the Hg° removal only occurred within the first 30 minutes. Afterwards, the removal efficiencies sharply decreased and there was no Hg° removed after 60 min. With the addition of Pd onto the Fe_3_O_4_–NH_2_, the Hg° removal ability was significantly enhanced. For example, the Fe_3_O_4_–NH_2_–Pd with 1% Pd loaded on Fe_3_O_4_–NH_2_ could remove approximately 45.7% of the Hg° after 8 h. A higher Hg° removal efficiency for the Fe_3_O_4_–NH_2_–Pd sorbent was observed when 5 wt.% Pd was loaded. However, further increase of the Pd content, such as 10 wt.% Pd in the Fe_3_O_4_–NH_2_–Pd, resulted in a slight decrease of the Hg° removal efficiency. Therefore, Fe_3_O_4_–NH_2_–Pd sorbent containing 5 wt.% Pd were used in the following experiments to study the impact of temperature on Hg° removal and its regeneration performance.

### Effect of temperature on Hg° removal over the magnetic sorbents

**Figure 2:**
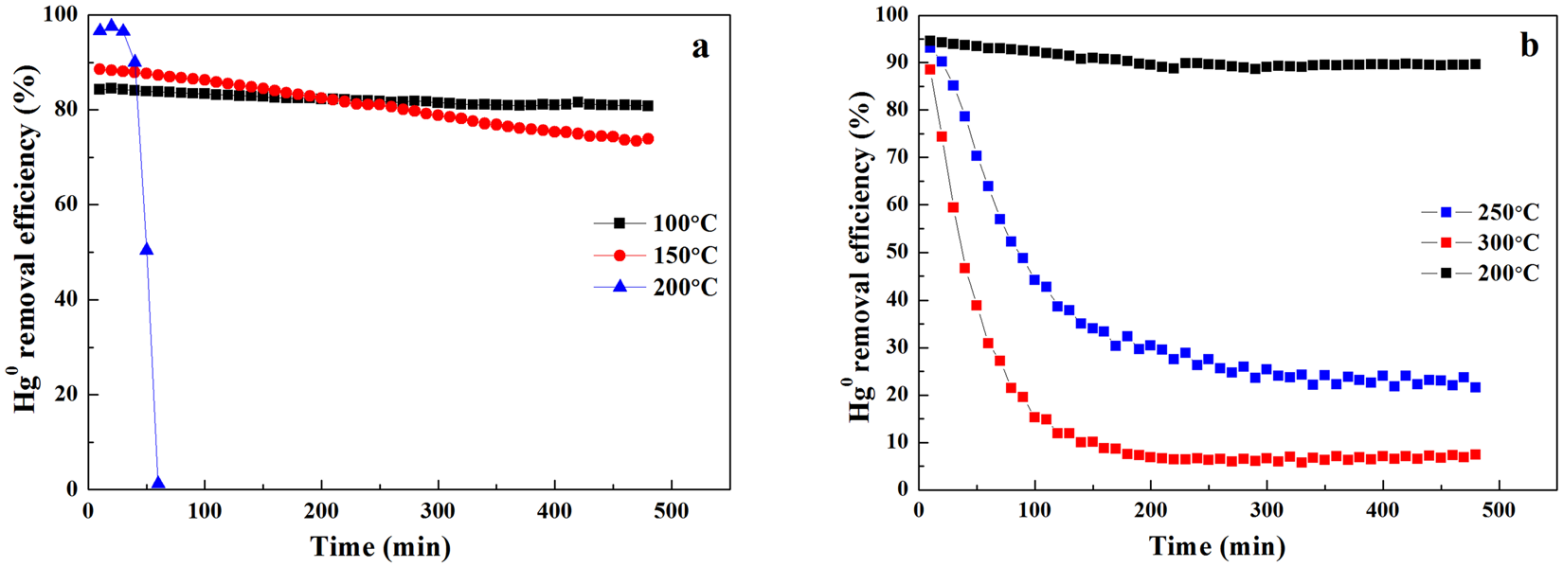
Efficiencies of Hg^0^ removal over the Fe_3_O_4_–NH_2_ (**a**) and Fe_3_O_4_–NH_2_–Pd (**b**) sorbents at different temperatures.

This section shows the effects of temperatures on removing Hg° for the sorbents of Fe_3_O_4_–NH_2_ and Fe_3_O_4_–NH_2_–Pd with 5wt.% Pd loaded. The results are shown in Fig. 2. It was observed that the Hg° removal efficiency for the Fe_3_O_4_–NH_2_ sorbent can remain above 80% for 500 min at 100 °C, as seen in Fig. 2a. When the temperature increased to 150 °C, the Hg° removal efficiency decreased from 88% to 73% within 500 min. At 200 °C, the Hg° removal efficiency for the Fe_3_O_4_–NH_2_ sorbent sharply decreased from 96% to 0 within 60 min. These results implied that at low temperature, Fe_3_O_4_–NH_2_ sorbent shows a good performance in removing Hg°.

Compared to the Fe_3_O_4_–NH_2_ sorbent, when the Pd was loaded, the Fe_3_O_4_–NH_2_–Pd sorbent showed a significantly increased capacity in capturing Hg° from the simulated coal derived fuel gas at a higher temperature, as seen in Fig. 2b. At 200 °C, the Fe_3_O_4_–NH_2_–Pd sorbent can remove more than 90% of the Hg°, and the removal efficiency did not decrease within the tested time 500 min. With increasing temperature, the removal efficiency significantly decreased, such as 250 and 300 °C. However, the removal efficiency was still higher than that of the Fe_3_O_4_–NH_2_ sorbent at 200 °C. Therefore, it can be concluded that the addition of Pd can dramatically improve the Hg° removal performance of the Fe_3_O_4_–NH_2_ sorbent, and the Fe_3_O_4_–NH_2_–Pd sorbent can be used at a higher temperature for capturing Hg°. On the basis of the different active temperatures for removing Hg°, it can thus be inferred that the Fe_3_O_4_–NH_2_ and Fe_3_O_4_–NH_2_–Pd sorbents could remove Hg° at different paths.

**Figure 3:**
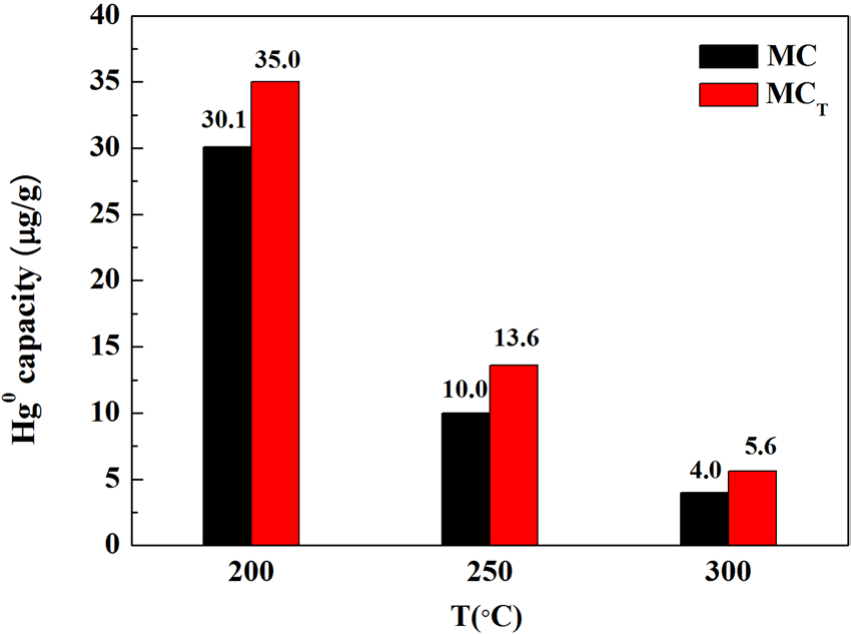
Mercury capacity and theoretical adsorption capacity of mercury of Fe_3_O_4_-NH_2_-Pd at different temperatures.

Figure 3 summarizes the experimental tested mercury capacity (MC) and the theoretical mercury capacity (MC_T_) of the Fe_3_O_4_–NH_2_–Pd sorbents. Clearly, it can be seen that the mercury capacities decreased with temperature. Compared to the theoretical mercury capacities, it can be seen that the MC obtained at three different temperatures were all lower than corresponding MC_T_. For instance, at 200 °C, the experimental mercury capacity was 30.1 μg/g, while the theoretical value is 35.0 μg/g. This is because the experimental mercury capacity (MC) was based on the measurements of the pyrolysis accessories of the mercury analyzer that can detect all the mercury species. By contrast, the theoretical mercury capacity (MC_T_) was calculated on the basis of the curve of the Hg° removal efficiency that was generated based on the detected Hg° by the online mercury analyzer. The online mercury analyzer can only detect the Hg° species in the gaseous phase because of its measurement limitation. Therefore, the difference between MC and MC_T_ was considered the escaping oxidation state of mercury that were produced during removing Hg°. Such small difference, such as less than 5 μg/g, indicated that the amount of the escaping oxidation state of mercury was very limited and the majority of the mercury were absorbed as Hg° by the Fe_3_O_4_–NH_2_–Pd sorbent.

### Performance of the regenerated Hg° sorbent

**Figure 4:**
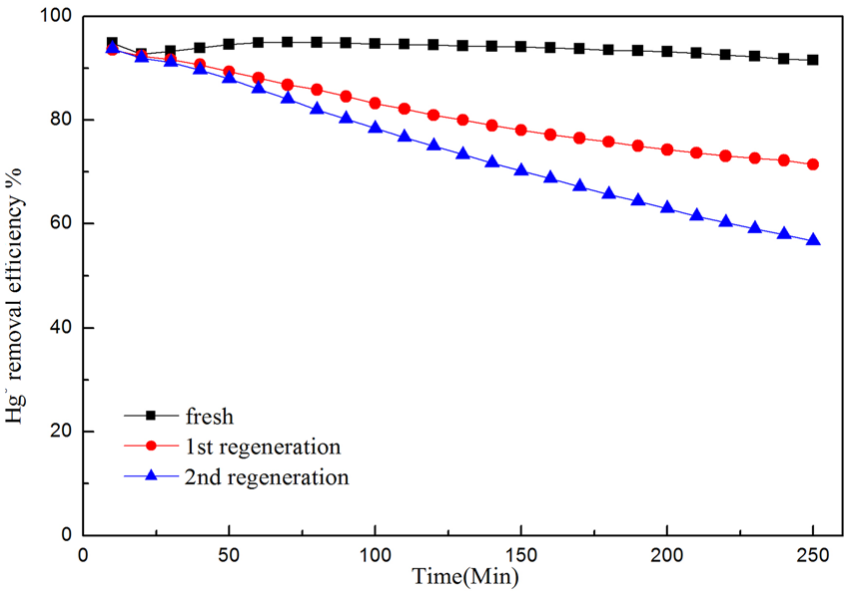
Efficiencies of Hg^0^ removal over Fe_3_O_4_-NH_2_-Pd along with two regeneration cycles.

In practice, the utilization of this mercury sorbent could be improved if it can be recycled by regeneration. Therefore, the performance of the multi-regenerated sorbent was included in this work. It has been found that the used magnetic materials can be easily regenerated by the external magnetic force. The used sorbent had been regenerated at 300 °C under N_2_ for 2 hrs before it was used for the second Hg° removal test. The results are shown in Fig. 4. It can be seen that, for the first regenerated sorbent, the Hg° removal efficiency dropped from 96% to only 72% after 250 min. By contrast, it dropped linearly to 56% for the second regenerated sorbent with the same period of time. The unknown strategy of how to improve the performance of the regenerated Fe_3_O_4_–NH_2_–Pd sorbent will be an angle for the future work.

### Recoverability of the as-synthesized Fe_3_O_4_–NH_2_ and Fe_3_O_4_–NH_2_–Pd sorbents

**Figure 5:**
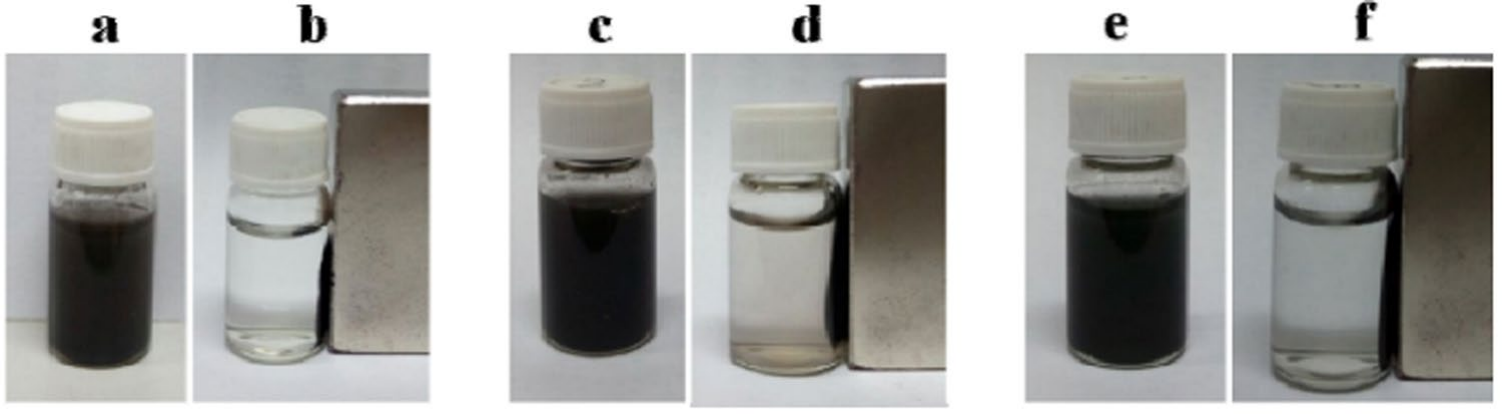
Photographs: (a) Fe_3_O_4_–NH_2_ in water, (b) Fe_3_O_4_–NH_2_ being pulled by magnet, (c) Fe_3_O_4_–NH_2_–Pd in water, (d) Fe_3_O_4_–NH_2_–Pd being pulled by magnet, (e) used Fe_3_O_4_–NH_2_–Pd in water and (f) used Fe_3_O_4_–NH_2_–Pd being pulled by magnet.

The recoverabilities of the as-synthesized Fe_3_O_4_–NH_2_ and Fe_3_O_4_–NH_2_–Pd magnetic nanocomposite were also investigated. It can be seen from Fig. 5a and c that the Fe_3_O_4_–NH_2_ and Fe_3_O_4_–NH_2_–Pd magnetic nanoparticles can disperse in water to form a black suspension solution. They can be pulled to the sidewall from the solution by applying a magnet besides the vial (Fig. 5b and d). Figures 5e and f show that Fe_3_O_4_–NH_2_–Pd sorbent had excellent magnetic properties even after two regeneration cycles of removal of Hg°, which was convenient for separation and recovery.

### Stabilization of the Hg in the sorbent phase

The stabilization of the Hg in the sorbent was tested based on the following experiments. Firstly, the Hg content of the used Fe_3_O_4_–NH_2_–Pd sorbent after two days washing by water is 30.3 μg/g, corresponding to the data of 30.1 μg/g in Fig. 3. Also, the Hg content in the sorbents after being stored for more than one year at normal pressure and temperature is 29.7 μg/g, therefore, the Hg in the adsorbed phase can be considered to be stable.

### Characterization of Fe_3_O_4_–NH_2_ and Fe_3_O_4_–NH_2_–Pd sorbents

#### Analysis of FTIR

**Figure 6:**
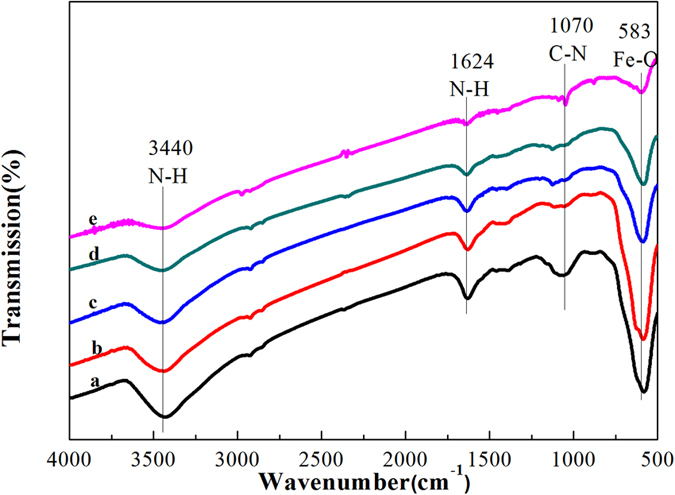
FT-IR spectra of (a) fresh Fe_3_O_4_-NH_2_, (b) fresh Fe_3_O_4_-NH_2_–Pd, (c) Fe_3_O_4_-NH_2_ after Hg^0^ removal at 100 °C, (d) Fe_3_O_4_-NH_2_–Pd after Hg^0^ removal at 200 °C and (e) Fe_3_O_4_-NH_2_–Pd after two regeneration cycles.

The existence of the amine functionalization was proved by the Fourier transform infrared (FTIR) spectra of the Fe_3_O_4_-NH_2_ and Fe_3_O_4_-NH_2_–Pd sorbents before and after the Hg° removal measurements. Figure 6 indicates that a strong peak at 583 cm^−1^ was assigned to the vibration of Fe-O bonds, which demonstrated the existence of the iron oxides. The peaks at 1070 cm^−1^, 1624 cm^−1^ and 3440 cm^−1^ correspond to C–N stretching vibration, N–H deformation vibration and N–H stretching vibration^29^, indicating the existence of the -NH_2_ group on the Fe_3_O_4_-NH_2_ sorbent. Therefore, it can be confirmed that the magnetic nanocrystals had been functionalized with amino groups in the synthetic process. There was no distinct variation after immobilization of palladium on the Fe_3_O_4_-NH_2_ surface except that the peak intensity became slightly weak. The FTIR spectrum of the used Fe_3_O_4_-NH_2_ (line c) and Fe_3_O_4_-NH_2_–Pd (line d) sorbents were shown. It was found that the signals (curves) did not show significantly change after the Hg° removal except for the peak intensity. The weak peak intensity of the Fe_3_O_4_-NH_2_–Pd after two regenerations (line e) indicated that the structure of Fe_3_O_4_-NH_2_–Pd changed partly. This is considered one of the reasons of the lower Hg removal efficiencies for the regenerated sorbent.

### Analysis of SEM and TEM

**Figure 7:**
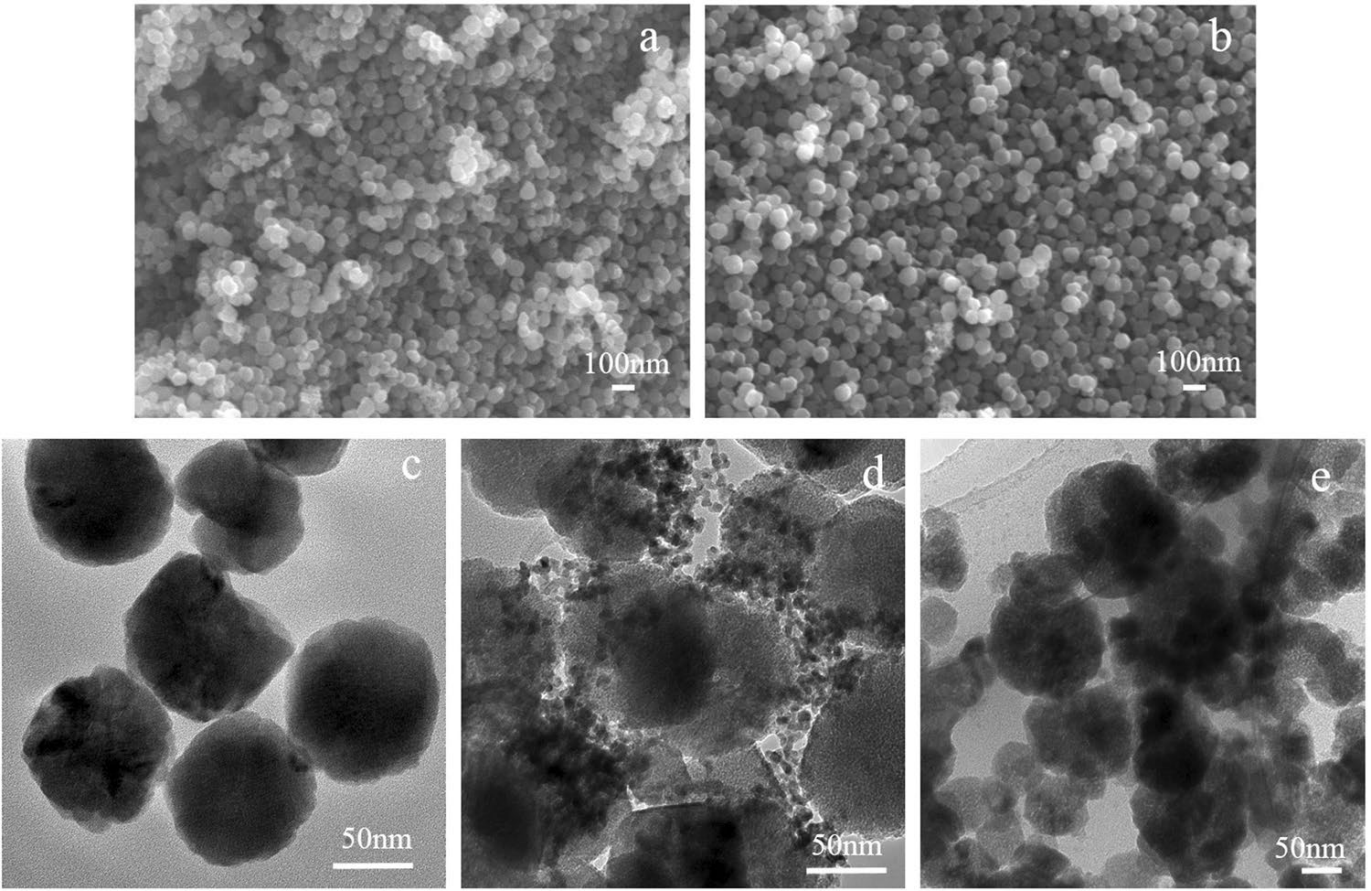
SEM images of (**a**) fresh Fe_3_O_4_–NH_2_ and (**b**) fresh Fe_3_O_4_–NH_2_–Pd; TEM images of (**c**) fresh Fe_3_O_4_-NH_2_, (**d**) fresh Fe_3_O_4_–NH_2_–Pd and (**e**) Fe_3_O_4_–NH_2_–Pd after two regeneration cycles.

The morphology and crystallography of the as-synthesized sorbents were characterized by SEM, and the structure was clearly revealed by TEM images. Figure 7a and b indicate the Fe_3_O_4_-NH_2_ and Fe_3_O_4_–NH_2_–Pd particles were regular sphere and the diameters of those spheres were less than 100 nm. There was no Pd nanoparticles identified according to the SEM images, which provides a clear evidence that the Pd nanoparticles had successfully loaded onto the surface of the Fe_3_O_4_^-^NH_2_ nanoparticles.

The TEM image in Fig. 7c shows that the synthesized Fe_3_O_4_-NH_2_ nanoparticles were nearly monodisperse with an average diameter of 90 nm. Figure 7d shows that the Fe_3_O_4_–NH_2_–Pd showed the uniform TEM micrographs. The small Pd nanoparticles were highly dispersed around the surface of the magnetite. The overall morphology and the size of these particles did not vary obviously after the palladium was attached onto the surface of the magnetic nanoparticles. Besides, it can also be concluded that the particle size distribution of the Pd was centered within 7 nm. Figure 7e showed that the morphologies of the Fe_3_O_4_–NH_2_–Pd particles after two regenerations varied significantly compared to the fresh sorbent. It can be seen that the looser and larger aggregate structures were formed after two regeneration for the Fe_3_O_4_–NH_2_–Pd sorbents. Some Fe_3_O_4_ particles were crushed and the Pd particles were partly aggregated, this may be the second reason that leads to the low Hg° removal efficiency for the Fe_3_O_4_–NH_2_–Pd sorbents after two regenerations.

### Analysis of XRD

**Figure 8:**
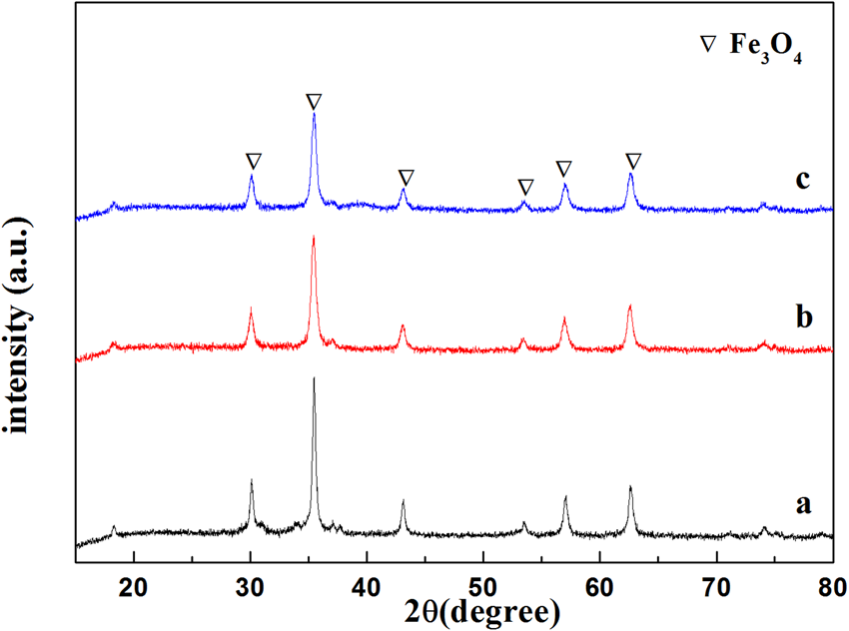
XRD patterns of (**a**) fresh Fe_3_O_4_-NH_2_, (**b**) fresh Fe_3_O_4_–NH_2_–Pd and (**c**) Fe_3_O_4_–NH_2_–Pd after two regeneration cycles.

The crystalline structures of the Fe_3_O_4_–NH_2_ and the Fe_3_O_4_–NH_2_–Pd were determined by the powder X-ray diffraction (XRD). As presented in Fig. 8, six characteristic diffraction peaks (2θ = 30.1, 35.5, 43.1, 53.5, 57.0 and 62.5°) can be clearly observed for Fe_3_O_4_-NH_2_ particles. The positions and relative intensities of all diffraction peaks matched well with those from the JCPDS card (75–1610) for magnetite. These peaks were sharp and strong, indicating the products were well crystallized. However, no diffraction peaks for the Pd species were found from the XRD patterns of the fresh Fe_3_O_4_–NH_2_–Pd sorbent, indicating that Pd species highly dispersed^30^ on the surface of Fe_3_O_4_–NH_2_–Pd nanoparticles after the reduction with KBH_4_, which was coincident with the results of TEM in Fig. 7d. The characteristic diffraction peaks of Fe_3_O_4_–NH_2_–Pd after two regeneration cycles were slightly weak compared to those of the fresh counterpart.

### Analysis of XPS

**Figure 9:**
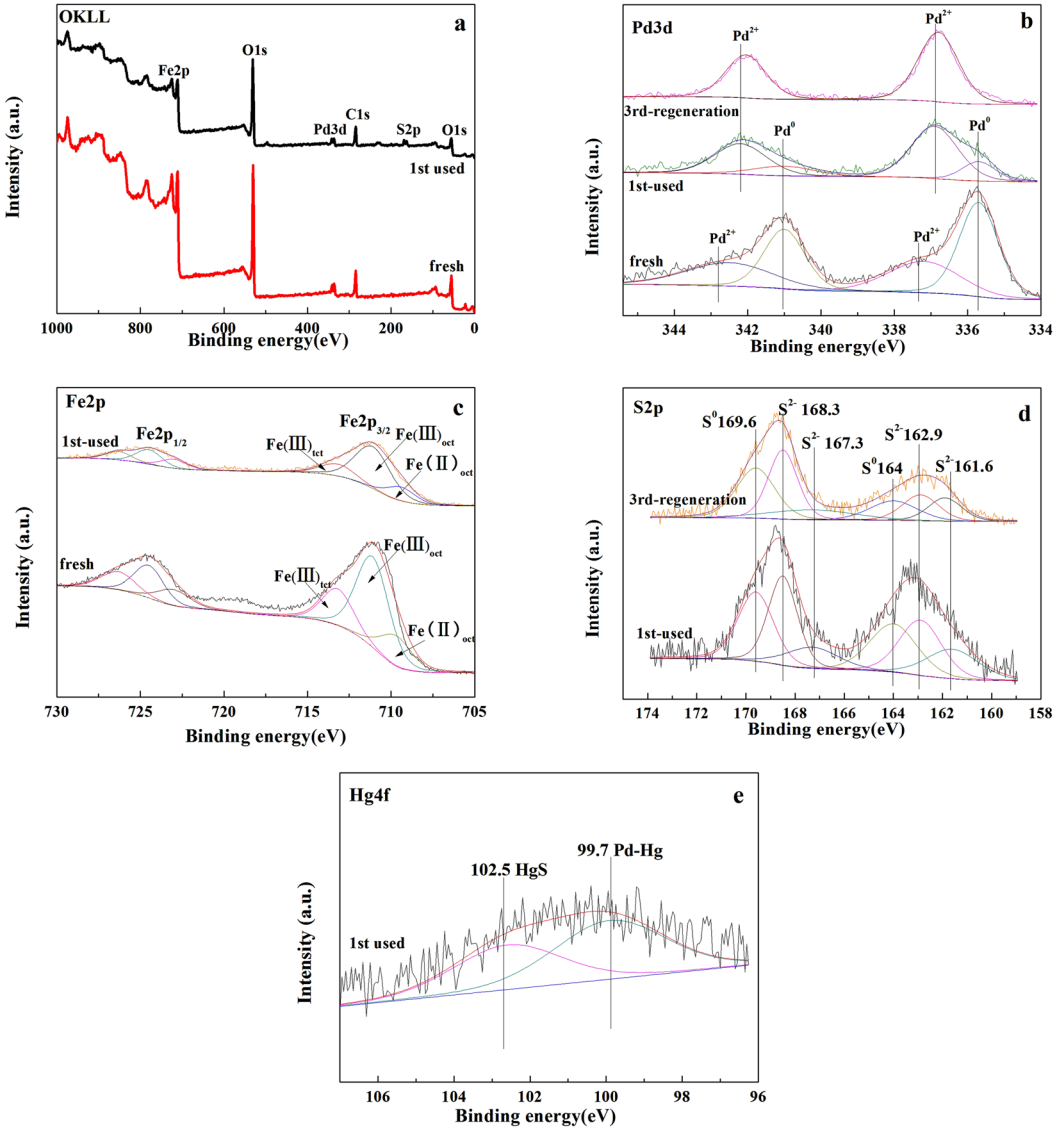
XPS spectra of Fe_3_O_4_–NH_2_–Pd sorbents: (**a**) survey scan and high-resolution scan of (**b**) Pd3d, (**c**) Fe2p, (**d**) S2p and (**e**) Hg4f.

XPS spectra of both survey and high-resolution scans for the key elements on Fe_3_O_4_–NH_2_–Pd surface were used to determine element valence of the sorbents before and after the Hg° removal, as seen in Fig. 9. The peaks corresponding to oxygen, iron, palladium and carbon were found in the survey spectra (Fig. 9a). Especially, the peaks assigned to sulfur were also observed in the used Fe_3_O_4_–NH_2_–Pd (Fig. 9a). It indicated that some sulfur species were absorbed on the surface of the Fe_3_O_4_–NH_2_–Pd.

XPS spectra of Pd *3d* were showed in Fig. 9b. The binding energy of 335.75 eV (Pd *3d*_5/2_) and 341 eV (Pd *3d*_3/2_) for both the fresh and used Fe_3_O_4_–NH_2_–Pd were assigned to Pd°^31^. According to the literature^32^, the binding energy of Pd° (Pd *3d*_5/2_) was 335.1 eV. However, it is found in this study that the Pd *3d*_5/2_ peaks shifted to higher values as a result of the presence of the surrounding positively charged ammonium groups. It implied the existing of –NH_2_ group can stable Pd on the surface of the Fe_3_O_4_–NH_2_ nanoparticles. The unreacted Pd° and Pd-Hg amalgam formed during the Hg° removal can be found in the used sample^8^.

The binding energy of 337.4 eV, 336.9 eV (Pd *3d*_5/2_) and 342.7 eV, 342.2 eV (Pd *3d*_3/2_) could assign to Pd^2+^. The Pd *3d*_3/2_ peaks also shifted to higher values due to the presence of ammonium groups^33^. Pd^2+^ species in the fresh sorbent could be assigned to PdO that were from the oxidation of the Pd° by the lattice oxygen of Fe_3_O_4_. PdO can react with H_2_S to form PdS. Pd^2+^ on the surface of the used and the regenerated sorbents may be assigned to PdS.

The intensity of the XPS spectrum peak reflects the content of the surface atom^34^. Table 1 showed the key elements contents on the surface for the fresh and used Fe_3_O_4_–NH_2_–Pd based on the results of the XPS spectra. The ratio of Pd°/Pd on the surface of the fresh Fe_3_O_4_–NH_2_–Pd was 71.17%, indicating that the elemental Pd was the main composition of the fresh sorbent. It was deduced from the Pd^2+^/Pd ratio on the surface of the used Fe_3_O_4_–NH_2_–Pd sorbents (79.08%) that the Pd^2+^ species was the main compositions after the removal of Hg°. The ratio of the Pd°/Pd on the surface for the used Fe_3_O_4_–NH_2_–Pd decreased to 20.92%, accordingly. It indicated that the elemental Pd on the surface of the fresh sorbent was greatly oxidized to Pd^2+^ species such as PdO or PdS during the Hg° removal. It was found in Fig. 9b that only Pd^2+^ of Pd existed in Fe_3_O_4_–NH_2_–Pd after three regeneration cycles while the Hg° removal efficiency sharply decreased for the Fe_3_O_4_–NH_2_–Pd after two regeneration cycles (showed in Fig. 4c), it also demonstrated that Pd° was the main active component.

**Table 1:** Concentrations of elements on the surface of the fresh and used Fe_3_O_4_–NH_2_–Pd sorbents according to the XPS results.

Samples	Pd_sur_ (wt.%)	j	Pd^2+^/Pd (%)	Fe_sur_ (wt %)	Fe^3+^/Fe^2+^ (%)	O_sur_ (wt %)	S_sur_ (wt.%)	Hg_sur_ (wt.%)
fresh	0.21	71.17	28.83	3.45	4.36	91.82	—	—
used	0.16	20.92	79.08	2.34	3.99	91.80	0.58	0.027

Some variations of the relative abundance between Fe^2+^ and Fe^3+^ species before and after removing of Hg° were elucidated by Fe *2p* XPS spectra in Fig. 9c. The Fe *2p* peak consisted of octahedral Fe^2+^ (710 eV), octahedral Fe^3+^ (712 eV), and tetrahedral Fe^3+^ (714 eV) peaks^35^. These values were very close to those of magnetite (Fe_3_O_4_) reported in the literature^35^. The ratios of Fe^3+^/Fe^2+^ on the surface of Fe_3_O_4_–NH_2_–Pd sorbent before and after removing Hg° decreased from 4.36 to 3.99 (showed in Table 1). Our previous study^8^ showed that Fe_2_O_3_ can react with H_2_S to produce FeS and S_ad_ (3H_2_S + Fe_2_O_3_ → 2FeS + S_ad_ + 3H_2_O). This result suggested that Fe^3+^ could be reduced to Fe^2+^ by H_2_S. However, the magnetism of Fe_3_O_4_–NH_2_–Pd sorbent did not change obviously based on the results of recoverabilities of the used Fe_3_O_4_–NH_2_–Pd sorbents.

Figure 9d shows the XPS spectra of S *2p* on the surface of the used Fe_3_O_4_–NH_2_–Pd sorbent. The binding energy of 161.6 eV and 162.9 eV can be assigned to PdS^36^ and HgS^37^, respectively. The binding energy of 164.0 eV belonged to elemental S^38^. This result proved that H_2_S could react with lattice oxygen and Fe^3+^ in Fe_3_O_4_ to form FeS, elemental S and H_2_O. Afterwards, the S can react with Hg to form HgS which is considered the Hg removal reaction. However, the active temperature range of the reactions to produce HgS was very limited, being efficient only in the range of 60–140 °C^39^ since elemental S was volatized with the increasing temperature. This can explain that the high efficiency in Hg° removal over Fe_3_O_4_–NH_2_ sorbent at 100 °C and low efficiency at 150 °C and 200 °C. However, for Fe_3_O_4_–NH_2_–Pd sorbent, the active temperature range of the Hg° removal was enlarged because of the loading of Pd. Pd and Hg can react to generate Pd-Hg amalgam (showed in Fig. 9b), resulting in the removal of Hg°. The content of sulfur on the surface of the used Fe_3_O_4_–NH_2_–Pd sorbent was 0.58%, indicating that there are abundant sulfur species such as elemental S, HgS, PdS and FeS after of the Hg° removal.

Figure 9e presents the XPS spectra of Hg *4 f* on the surface of the used Fe_3_O_4_–NH_2_–Pd sorbent. Because the binding energy of Hg° (Hg *4e*_7/2_) was around 99.9 eV^40^, it can be inferred that the binding energy of 99.7 eV can be assigned to Pd-Hg amalgam. Also, that of 102.5 eV was consistent with Hg^2+^ compounds (HgS)^37^.

### Mechanism of the Hg° removal of Fe_3_O_4_–NH_2_–Pd sorbent

The experimental results indicated that the Fe_3_O_4_–NH_2_–Pd sorbent showed a good activity in removing Hg°. Based on the experimental result, the mechanism of the Hg° removal over Fe_3_O_4_–NH_2_–Pd sorbent could be discussed as follow: there were two paths in removing Hg° from coal derived fuel gas over the Fe_3_O_4_–NH_2_–Pd sorbent. The first one was the reaction of elemental Pd and Hg to form Pd-Hg amalgam (shown in Equation 1). The second one was the reaction of elemental Hg and S_ad_ to form HgS (Equation 3), and S_ad_ was produced by the oxidation of H_2_S by lattice oxygen in Fe_3_O_4_ (Equation 2). In addition, H_2_S can react with PdO to form PdS (Equation 4). For Fe_3_O_4_–NH_2_ sorbent, there was only one path to remove Hg°. That was the reaction of elemental S produced by the oxidation of H_2_S and Hg to form HgS. Because of elemental S can be volatized with the increase of temperature, the active temperature of the Hg° removal over Fe_3_O_4_–NH_2_ sorbent was as low as 150 °C. For Fe_3_O_4_–NH_2_–Pd, the active temperature of the Hg° removal was higher because that Pd and Hg can react to generate Pd-Hg amalgam.

1 $${\rm{Pd}}+{\rm{Hg}}\to {\rm{Pd}}-{\rm{Hg}}\,{\rm{amalgam}}$$

2 $${{\rm{H}}}_{2}{\rm{S}}+{{\rm{Fe}}}^{3+}+{\rm{O}}\to {{\rm{Fe}}}^{2+}+{{\rm{S}}}_{{\rm{ad}}}+{{\rm{H}}}_{2}{\rm{O}}$$

3 $${{\rm{S}}}_{{\rm{ad}}}+{\rm{Hg}}\to {\rm{HgS}}$$

4 $${{\rm{P}}{\rm{d}}{\rm{O}}+H}_{2}{S\to PdS+H}_{2}{\rm{O}}$$

## Conclusion

The magnetical Fe_3_O_4_–NH_2_–Pd sorbent was successfully synthesized using a facile one-pot template-free method combined with a metal adsorption-reduction procedure on the Fe_3_O_4_–NH_2_. The performances of the Hg° removal over the Fe_3_O_4_–NH_2_ and Fe_3_O_4_–NH_2_–Pd sorbents showed that the loaded Pd on the amine-functionalized magnetite nanoparticles could greatly enhance the efficiency in removing Hg^0^ at a higher operation temperature range between 200 and 300 °C. The Fe_3_O_4_–NH_2_–Pd sorbent with a 5% Pd loaded exhibited significantly high activity and stability in Hg^0^ capture, affording over 93% capture efficiency at 200 °C for more than 8 hrs. In term of the mechanism, it was considered that the Hg^0^ was removed on the Fe_3_O_4_–NH_2_–Pd sorbent by forming Pd-Hg amalgam and HgS. By contrast, the Hg^0^ was mainly converted as HgS over the Fe_3_O_4_–NH_2_ sorbent. With regard to the recyclable utilization, the used sorbents could be easily regenerated at 300 °C under N_2_ atmosphere. The activity in removing Hg^0^ for the regenerated Fe_3_O_4_–NH_2_–Pd sorbent slightly decreased, however, the magnetic properties after two regenerations were still stable. These findings proved that the Fe_3_O_4_–NH_2_–Pd sorbent could be considered as a recyclable candidate for the Hg^0^ removal from coal derived fuel gas.

## Method

Ferric chloride hexahydrate (FeCl_3_·6H_2_O), anhydrous sodium acetate (NaAc), ethylene glycol (EG), 1,6-hexanediamine and ethanol, potassium borohydride (KBH_4_), Pd(NO_3_)_2_, all chemicals were purchased form Aladdin Industrial Corporation and used as received without further treatment. De-ionized water was used throughout the experiments.

The Fe_3_O_4_–NH_2_ nanoparticles were prepared according to the reference^41^ and we do some modification on it. Typically, a solution of 6.5 g 1,6-hexanediamine, 2.0 g anhydrous sodium acetate and 1.2 g FeCl_3_·6H_2_O as a ferric source in 35 mL ethylene glycol was stirred at 50 °C to give a transparent solution. The solution was then transferred into a teflon-lined autoclave and then kept at 200 °C for 6 hrs. The magnetic nanoparticles were then rinsed with water and ethanol for several times to effectively remove the solvent and unbound 1, 6-hexanediamine, and then dried under vacuum at room temperature to obtain a black powder for further use. During each rinsing step, the nanoparticles were separated from the supernatant by using a magnetic force.

The Fe_3_O_4_–NH_2_–Pd nanoparticles were prepared according to the reported method^29^ with some modification. 0.5 g of synthesized Fe_3_O_4_–NH_2_ nanoparticles was placed in a 50 mL ethanol solution and then treated with ultrasonic for 0.5 hrs. This black suspension solution was mixed with 3.0 mM of a Pd(NO_3_)_2_ solution for 1 hrs with ultrasonic. Then the sorbent was reduced by an excess 0.1 M KBH_4_ aqueous solution. It was slowly dropped into the above mixture with stirring. The solid sorbent was separated by magnet and was washed by deionized water after 2 hrs of reduction. The sorbent was dried at 45 °C under vacuum. The Pd loading amounts in the sorbent ranged from 0 to 10 wt %. Finally, all samples were pressed for tableting and then sieved at 40–60 mesh.

The performance of Hg^0^ removal over the as-synthesized Fe_3_O_4_–NH_2_ and Fe_3_O_4_–NH_2_–Pd sorbents was detected by a fixed-bed reactor. It includes four parts: Hg generation, gas mixture, a reactor and an online mercury analyzer (Lumex RA-915 M + Zeeman, Lumex-Marketing JSC, Russia) as the detection system. Hg vapor was generated by a Hg permeation tube (Valco Instruments Company Inc., America) immersed in a constant water bath maintained at 35 ± 0.5 °C. Hg vapor was brought into the evaluation system using ultrahigh purity N_2_ as a carrier. The flow rate through the U tube was accurately controlled by a mass-flow controller. The simulated fuel gas consisted of 10 vol.% H_2_, 20 vol.% CO, 300 ppm H_2_S, 45 ± 3 μg/m^3^ Hg^0^ vapor, balancing gas N_2_ (470 ml/min) and carrier gas N_2_ (500 ml/min).

A total of 0.50 g sorbent was placed in the horizontal quartz reactor (5.0 mm of the inner diameter) and then packed with quartz wool to support the sorbent layer and avoid its loss. Subsequently, the simulated fuel gas was switched into the reactor at the desired temperature. Hg vapor concentrations at the inlet and outlet of the reactor containing sorbents were monitored using a Lumex mercury analyzer. The removal efficiency (η) of Hg^0^ was used to evaluate the performance of the sorbents for the capture of Hg^0^ from coal derived fuel gas. η is calculated by the following formula:

5 $$\eta ( \% )\,=\,\frac{{{\rm{C}}}_{{\rm{0}}}{-C}_{{\rm{1}}}}{{{\rm{C}}}_{{\rm{0}}}}\times \mathrm{100} \% $$

where C_0_ and C_1_, μg/m^3^ or ppm, are the concentrations of Hg^0^ in the feed and effluent gases, respectively.

Mercury content of the sorbent after evaluating is defined as mercury capacity (MC) and it can be directly detected by the pyrolysis accessories (PYRO-915+) of mercury analyzer. All the mercury species can be detected by the pyrolysis accessories of mercury analyzer. Theoretical adsorption mercury capacity (MC_T_) of the sorbents can be calculated by the curve of the Hg^0^ removal efficiency, the formulas is showed as bellow:

6 $$M{C}_{T}\,(\mu g/g)\,=\,\sum {\eta }_{i}\frac{{C}_{0}{Q}_{i}{t}_{i}}{G\times 1000}$$

where ***η***_***i***_ is the mercury removal efficiency at ***t***_***i***_ which is the adsorption time in the ***i*** reactive time (min), ***C***_***0***_ is the initial concentration (μg/m^3^) of Hg^0^ in the feed gas, ***Q***_***i***_ is the flow rate of coal derived fuel gas, ***G*** is the weight of sorbent in the reactor (g).

After the mercury removal test, the used sorbents were regenerated by heating to 300 °C in pure N_2_ carrier gas for 2 hrs. Several capture–regeneration cycles were conducted to evaluate the regeneration performance of Fe_3_O_4_–NH_2_–Pd sorbent.

The fresh and used Fe_3_O_4_–NH_2_ and Fe_3_O_4_–NH_2_–Pd sorbents were determined by Fourier transform infrared (FTIR, Bruker Vertex 70) and the scan range is from 4000 cm^−1^ to 400 cm^−1^.

Morphology and particle dispersion of the as-synthesized Fe_3_O_4_–NH_2_ and Fe_3_O_4_–NH_2_–Pd were investigated by scanning electron microscopy (SEM) (Cam scan MV2300). The chemical compositions of the synthesized nanostructures were measured by EDS performed of SEM. Transmission electron microscopy (TEM) images were obtained on a Hitachi H-800 transmission electron microscope with an accelerating voltage of 200 kV.

X-ray diffraction (XRD) was employed to investigate the crystal structures of the synthesized sorbents. The instrument was a Rigaku Miniflex 600 diffractometer, fitted with a nickel-filtered Cu K α radiation source operating at a voltage of 40 kV and 100 mA. The scan rate was 8°/min in the range from 15° to 80°.

X-ray photoelectron spectroscopy (XPS) surface analysis was conducted to determine the elemental speciation and concentration on the surface of Fe_3_O_4_–NH_2_–Pd sorbents, using an ESCALAB 250 spectrometer (VG Scientific Ltd., UK) equipped with an Al Kα source (1486.6 eV, 150 W). Energy calibration was performed using C 1 s peak at 284.6 eV. No smoothing routine of data was applied to analyze the results.
